# Effectiveness and Cost-Effectiveness of Receiving a Hearing Dog on Mental Well-Being and Health in People With Hearing Loss: Protocol for a Randomized Controlled Trial

**DOI:** 10.2196/15452

**Published:** 2020-04-17

**Authors:** Lucy Stuttard, Catherine Hewitt, Caroline Fairhurst, Helen Weatherly, Simon Walker, Francesco Longo, Jane Maddison, Philip Boyle, Bryony Beresford

**Affiliations:** 1 Social Policy Research Unit Department of Social Policy and Social Work Alcuin B Block, University of York York United Kingdom; 2 York Trials Unit Department of Health Sciences University of York York United Kingdom; 3 Centre for Health Economics University of York York United Kingdom

**Keywords:** randomized controlled trial, hearing loss, qualitative research, economics, assistance dog

## Abstract

**Background:**

People with hearing loss, particularly those who lose their hearing in adulthood, are at an increased risk of social isolation, mental health difficulties, unemployment, loss of independence, risk of accidents, and impaired quality of life. In the United Kingdom, a single third-sector organization provides hearing dogs, a specific type of assistance dog trained to provide sound support to people with hearing loss. These dogs may also deliver numerous psychosocial benefits to recipients. This has not previously been fully investigated.

**Objective:**

The study aims to evaluate the impact of a hearing dog partnership on the lives of individuals with severe or profound hearing loss.

**Methods:**

A 2-arm, randomized controlled trial will be conducted within the United Kingdom with 162 hearing dog applicants, aged 18 years and older. Participants will be randomized 1:1 using a matched-pairs design to receive a hearing dog sooner than usual (intervention arm: arm B) or to receive a hearing dog within the usual timeframe (comparator arm: arm A). In the effectiveness analysis, the primary outcome is a comparison of mental well-being 6 months after participants in arm B have received a hearing dog (arm A have not yet received a hearing dog), measured using the Short Warwick Edinburgh Mental Well-Being Scale. Secondary outcome measures include the Patient Health Questionnaire-9, Generalized Anxiety Disorder-7, and Work and Social Adjustments Scale. An economic evaluation will assess the cost-effectiveness, including health-related quality-adjusted life years using the EuroQol 5 Dimensions and social care–related quality-adjusted life years. Participants will be followed up for up to 2 years. A nested qualitative study will investigate the impacts of having a hearing dog and how these impacts occur.

**Results:**

The study is funded by the National Institute for Health Research’s School for Social Care Research. Recruitment commenced in March 2017 and is now complete. A total of 165 participants were randomized. Data collection will continue until January 2020. Results will be published in peer-reviewed journals and at conferences. A summary of the findings will be made available to participants. Ethical approval was received from the University of York’s Department of Social Policy and Social Work Research Ethics Committee (reference SPSW/S/17/1).

**Conclusions:**

The findings from this study will provide, for the first time, strong and reliable evidence on the impact of having a hearing dog on people’s lives in terms of their quality of life, well-being, and mental health.

**Trial Registration:**

International Standard Randomised Controlled Trial Number Registry ISRCTN36452009; http://www.isrctn.com/ISRCTN36452009

**International Registered Report Identifier (IRRID):**

DERR1-10.2196/15452

## Introduction

### Background and Rationale

Around 5% of the world’s population currently experience disabling hearing loss, and this is estimated to nearly double by 2050 [1]. In the United Kingdom (UK), 1 in 6 adults are affected by hearing loss, and about 1% of the adult population is severely or profoundly deaf. For this latter population, hearing aids offer little benefit [2]. Adults with hearing loss, particularly those who acquired hearing loss in adulthood, are at a risk of adverse outcomes, including social isolation, emotional distress, mental health difficulties, unemployment, dependence, increased risk of accidents, and impaired quality of life [3-10]. Hearing loss is also associated with cognitive decline and an increased risk of dementia [11,12]. People experiencing hearing loss would like access to services, equipment, and assistive techniques that support mental well-being [13], activities of daily living, and the best quality of life [4]. In cases where people are unable to benefit from hearing aids [2], the focus shifts to adaptation of hearing loss and the prevention (or minimization) of adverse outcomes. A hearing dog is one of the support options available [14].

### About Hearing Dogs

In the United Kingdom, just one organization, Hearing Dogs for Deaf People [15] trains and provides hearing dogs to UK accredited assistance dog standards [16]. A hearing dog’s specialist training means that it can alert a deaf person to a range of everyday sounds (eg, cooker timers and alarm clocks), some of which support communication with others (eg, telephone calls, doorbells, and their name being called) and personal safety (eg, smoke alarms and baby cry monitors). A hearing dog lives with its deaf recipient and, in contrast to a pet dog, is legally permitted (under the Equality Act 2010) to accompany the recipient into service settings (eg, shops, pubs, and aircraft).

Once an application to Hearing Dogs for Deaf People is accepted, a client advisor liaises with the applicant over 3 to 6 months to create a detailed understanding and written *profile* of their lifestyle and hearing dog–relevant needs. This *profile* is then released to the hearing dog training staff who liaise with the client advisor to identify *matches* between an applicant and the pool of hearing dogs coming toward the end of their training (aged approximately 18 months old). It can take between 1 and 3 years for a hearing dog to be provided to an applicant depending on the complexity of their needs. During the period of introducing the hearing dog to the recipient and the dog settling in their home, a series of strategies ensure the hearing dog attaches exclusively to the recipient. Apart from when the recipient is exercising the dog, the hearing dog wears a distinctive *hearing dog jacket* when taken outside the home. Hearing Dogs for Deaf People refers to the arrangement of a hearing dog and recipient as a *hearing dog partnership*. Each recipient is allocated a partnership instructor for ongoing partnership support in their local community. Initially, partnership instructors visit regularly to offer bespoke advice and support, tapering to a minimum annual visit once the partnership is established, although recipients can still contact them for support at any time and attend community activities, events, and workshops. Routine visits ensure the recipient is maintaining the dog’s welfare, standard of general behavior, and sound work skills training, thus providing an opportunity for the recipient to request advice and support on these issues or other areas of concern. A hearing dog partnership can last up to 10 years, after which the dog is retired.

Hearing Dogs for Deaf People report that the cost of training a dog and creating and supporting a partnership throughout the dog’s working life is around UK £40,000 (US $52,000) [17].

Although Hearing Dogs for Deaf People retain ownership of their hearing dogs and cover the costs described above, recipients are responsible for covering day-to-day costs such as food, routine veterinary care, and insurance.

### Existing Evidence

A literature review of evaluations of assistance dogs was conducted to make the case for this research (internal report by Baxter and Beresford [18]). This review identified three studies that had evaluated hearing dog partnerships [19-21] and a further two with mixed samples of individuals with a hearing or mobility impairment [22,23]. These studies indicated that there could be psychological, social, and health benefits of an assistance dog, but the study designs were weak, eg, before and after studies and nonrandomized comparative studies, often with small samples. Only two studies, both mixed samples of individuals with a hearing or mobility impairment, considered the cost implications of a hearing dog. All studies apart from one [19] were conducted in the United States and Canada, where the findings may not be directly transferable to the UK setting. Since the publication of this review, two further studies looking at the impact of hearing dogs on quality of life have been published [24,25]. One of them was a UK study employing a nonrandomized design to compare outcomes for recipients of hearing dogs and mobility dogs to those waiting to receive one [24]. Some improvements in quality of life were reported, but the study had a poor response rate, and the representativeness of the surveyed sample with respect to the population of hearing dog applicants was unclear. The other was a longitudinal study conducted in Sweden of outcomes for 55 dog owners before and after their companion dog was trained to perform as an assistance dog [25]. The majority of these dogs were trained to become mobility (n=30) or diabetes assistance dogs (n=20), with only 3 of them being trained to become hearing dogs. Improvements in quality of life, well-being, and levels of physical activity were reported, but there was no comparator group.

### Design

The study will comprise a single-center, superiority randomized controlled trial (RCT) with nested economic and qualitative evaluations. In developing the protocol, the research team worked closely with Hearing Dogs for Deaf People to ensure the design fits with standard processes for creating hearing dog partnerships, the highly variable time it takes to match an applicant with a dog, and Hearing Dogs for Deaf People’s commitment to create a partnership within 3 years of application.

### Objectives

The overall aim of the study is to evaluate the impact of a hearing dog partnership on the lives of individuals with severe or profound hearing loss.

The study objectives are as follows:

- To determine the impact of a hearing dog partnership on mental well-being, as measured by the Short Warwick-Edinburgh Mental Well-Being Scale (SWEMWBS) [26], 6 months post receipt of a hearing dog, compared with applicants who have not yet received their hearing dog.
- To determine the impact of a hearing dog partnership on secondary outcomes of impairment in functioning, anxiety, depression, and health-related and social care–related quality of life 6 months postreceipt of a hearing dog.
- To conduct a nested economic evaluation to investigate the cost-effectiveness of hearing dogs.
- To conduct a nested longitudinal qualitative study to describe the impacts of a hearing dog on recipients’ lives, and the mechanisms by which these impacts occur.
- To gather initial data on the long-term outcomes of having a hearing dog.

## Methods

### Study Setting

The setting is Hearing Dogs for Deaf People, the only organization accredited to provide hearing dogs to UK residents. Research participants are first-time applicants for a hearing dog who may reside anywhere in the United Kingdom.

#### Inclusion Criterion

The inclusion criterion is first-time applicants for a hearing dog, aged 18 years and older.

#### Exclusion Criteria

The exclusion criteria include the following:

- Individuals aged 17 years or younger.
- Individuals requiring a dog who can provide sound and vision support.
- Individuals who are replacing a retiring hearing dog.
- Individuals with a learning disability (indicated by the use of a proxy during the application process).
- Individuals still awaiting a hearing dog but whose application is at a stage past the point where randomization could take place. These applicants will have the opportunity to complete the research materials and form a part of an exploratory longitudinal cohort study.

### Intervention

The intervention includes the receipt of a hearing dog specifically matched and trained to support the needs of the applicant. The comparator includes not having a hearing dog. Practices regarding cessation of a hearing dog partnership should align with the standard Hearing Dogs for Deaf People protocols. Outside of the intervention, participants should receive care as usual.

### Recruitment

Recruitment to the trial took place between March 2017 and March 2018. Hearing Dogs for Deaf People will screen all applications during this period. Typically, the charity receives over 200 applications annually. For those fulfilling the study eligibility criteria, Hearing Dogs for Deaf People will post a study recruitment pack (including a participant information sheet, consent form, contact preferences form, study questionnaire, and reply-paid envelope). Applicants wishing to participate can choose to complete the study materials on paper or on the Web in either English or British Sign Language (BSL) via Qualtrics, a Web-based survey platform. These will be returned directly to the research team. In the case of nonresponse, Hearing Dogs for Deaf People will send up to two reminders (text message and email or post). Where possible, reasons for decline will be obtained. Applicants not recruited to the study will follow standard Hearing Dogs for Deaf People procedures and timelines.

### Randomization

Randomization will be conducted centrally by the York Trials Unit (YTU), using an allocation schedule generated in Stata v15. During their hearing dog assessment, study participants will be categorized by a senior practitioner within Hearing Dogs for Deaf People, with regard to their presenting needs, as follows:

- None: no remarkable or particular needs.
- Personal: predominantly personal needs, this might include particular health concerns or mobility.
- Environmental: predominantly environmental needs, this might include an inner city location or the presence of cats in the home.
- Both: significant personal and environmental needs.

When the profile of 2 individuals with the same categorization of need is completed, they will form a pair and be randomized together, one to each group using block randomization with a block size of 2:

- Arm A: matching with a hearing dog occurs within usual timelines or
- Arm B: matching accelerated, receive a hearing dog at least six months earlier than those in arm A.

The allocation sequence will be generated by the trial statistician (CF) who has no involvement in the recruitment of participants. As pairwise randomization is being employed, ie, the randomization of 2 participants at a time, allocation is concealed.

Participants will be blinded to their group allocation. Study team members who are actively involved in the administration of the trial will not be blinded.

### Follow-Up Data Collection

Follow-up questionnaires will be administered by the research team via post or email according to participants’ preferences. Postal, email, and text reminders and an incentive (£20 per data collection time point) will support retention to the study.

**Figure 1:**
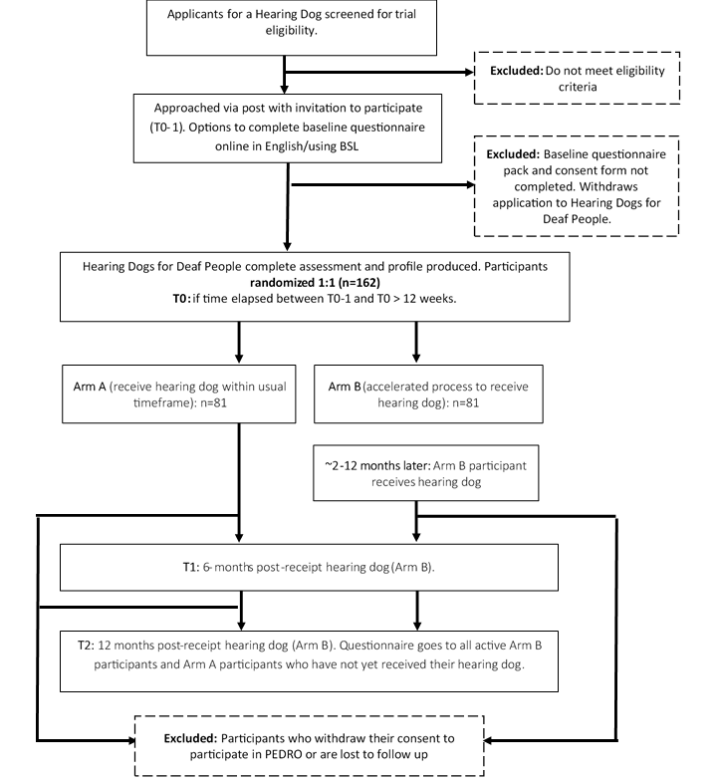
Flow of trial participants through partnerships between deaf people and hearing dogs.

Figure 1 presents the flow of study participants through the trial. T1 (6 months after the arm B participant has received their hearing dog) is the primary outcome time point. Arm B participants will also be followed up at 12 (T2), 18 (T3), and 24 (T4) months post receipt of a hearing dog. Arm A participants will only be followed up at T2 if they have not yet received their dog. Participants who withdraw their application or return their dog will continue to be contacted according to this schedule. In the case of arm B participants withdrawing, a dummy date of partnership will be created. Given the personalized nature of the intervention, some arm A participants may receive their dog before their arm B partner.

### Outcome Measures

The selection of outcome measures was informed by the existing literature and in consultation with Hearing Dogs for Deaf People and hearing dog recipients. All standardized measures are available in BSL, and these versions have satisfactory psychometric properties [27-29].

#### Primary Outcome for the Effectiveness Analysis

The SWEMWBS [26] comprises seven positively worded items related to psychological functioning with five response categories (*none of the time* to *all of the time*). Respondents indicate the response that best describes their experience over the last 2 weeks.

#### Primary Outcome for the Cost-Effectiveness Analysis

The EuroQol 5 Dimensions (EQ-5D-5L) is a measure of health-related quality of life capturing five domains: mobility, self-care, usual activities, pain/discomfort, and anxiety/depression [30]. Each domain is captured on a 5-point scale, with respondents reporting how they feel *today*.

#### Secondary Outcomes

The Work and Social Adjustments Scale (WSAS) measures impairment in functioning and comprises five items, one related to work and the remainder to social functioning [31]. Respondents rate their impairment on a 9-point scale (*not at all* to *very severely*).

Two measures of mental health will be used: the Patient Health Questionnaire (PHQ-9) and the Generalized Anxiety Disorder Assessment (GAD-7) [32,33]. Respondents rate how often they have experienced numerous mental health problems over the previous 2 weeks. Each item provides a 4-point response scale (*not at all* to *nearly every day*). Clinical cutoffs for BSL versions have been established [34].

Descriptive outcomes include the status of the hearing dog partnership (intact vs ceased); use of statutory, third sector, and private health and social care services in the past 4 weeks; and employment status.

The Hearing Dog Questionnaire [19] will be used to capture reports of problems associated with hearing impairment that a hearing dog is meant to alleviate: awareness of sounds (eg, smoke alarm and doorbell), concerns about security (in and outside the home), dependency on others, and being misunderstood when out in public. It comprises 11 items, and a 5-point scale captures the frequency at which these difficulties are experienced.

Study participants who receive a hearing dog during the trial will also be asked about the benefits and challenges of their hearing dog partnership using fixed response (eg, “How challenging did you find adjusting to having a hearing dog?” with four response options from *not at all challenging* to *very challenging*) and free-text response formats (eg, Please tell us briefly how your life has improved since having a hearing dog).

### Data Management

Data will be entered into SPSS 23 by someone who is independent of the data analysis. A random sample of 10% will be double entered to assess data quality. An error rate exceeding 5% will require investigation including an examination of the type of errors, eg, random vs systematic and potentially a double entry of the whole data set. Missing data from a scale will be managed using guidance from scale developers. For the SWEMWBS and WSAS, up to one missing item and, for GAD-7 and PHQ-9, up to two missing items will be replaced with the scale mean.

### Statistical Methods

#### Sample Size

There are limited published data on which to base a sample size for this trial. Therefore, we have taken a pragmatic approach and calculated a sample size that should be achievable within the study timescale. We will aim to approach at least 200 applicants, of which we expect approximately 180 to be eligible and of which 90.0% (162/180) will opt to join the study. Allowing for 20% attrition at T1, this will result in a trial sample at T1 of 128. This pragmatic sample size will provide 80% power at 5% significance to detect an effect size of 0.5 of a standard deviation on our primary outcome measure, the SWEMWBS. This is a moderate-to-large effect size.

#### Effectiveness Analysis

Trial analyses on the effectiveness of hearing dogs will be conducted on an intention-to-treat basis, including all participants in the arm to which they were randomized. Analyses will be conducted in SPSS 23 or later, using 2-sided statistical tests at the 5% significance level. The flow of participants through each stage of the trial will be presented in a Consolidated Standards of Reporting Trials diagram. The primary analysis will estimate the difference in the SWEMWBS scores at T1 between arms A and B using linear regression, adjusting for the baseline measure and the individual’s presenting needs (identified before randomization). The difference between arms in the SWEMWBS score at T1 and corresponding 95% CI will be presented. Model assumptions will be checked, and if they are in doubt, data transformations, alternative distributions, or nonparametric alternatives will be considered. The secondary outcomes (WSAS, PHQ-9, and GAD-7) will be analyzed in the same way.

Sensitivity analyses will investigate the robustness of the findings given any nonadherence to the protocol. Application withdrawals, placement cessations, and adverse events will be summarized for each group.

An exploratory analysis of outcomes at T2 will analyze the data using the same model as for the T1 with the caveat that there will be systematic missing data as T2 data are not collected from arm A participants who have received a hearing dog by this point. Further exploratory analyses will examine the longer-term outcomes collected for study participants where data collection at 18 (T3) and 24 (T4) months post receipt of a hearing dog is achieved. For these analyses, time will be added as a variable to the regression model.

To date, the properties of the Hearing Dog Questionnaire have not been tested to determine whether the items perform as a scale. We will use classical test theory [35] to determine whether it is appropriate to calculate a total or subscale scores. If this is not possible, we will present responses to the individual items.

The Statistical Analytical Plan will be uploaded to the trial registry.

### Economic Evaluation

An economic evaluation will be conducted to determine whether hearing dogs are value for money. It will be undertaken from multiple perspectives to inform value for money considerations for different potential decision makers. Perspectives will include (1) a voluntary sector perspective (including costs to Hearing Dogs for Deaf People and of volunteers); (2) a social care perspective (considering that costs of hearing dogs could fall on social care budgets); (3) a health care perspective (considering the costs of hearing dogs could fall on health care budgets); (4) a public sector perspective (considering impact on both social and health care budgets); and (5) a broader perspective considering costs to the voluntary sector, social care, health care, and costs incurred by the recipients of hearing dogs. The choice of outcome will be perspective dependent, choosing the most relevant outcome(s) for each decision maker, with social care quality-adjusted life years (SC-QALYs) and health quality-adjusted life years (H-QALYs) being key outcomes to consider. To determine cost-effectiveness, incremental costs and units of effect will be compared with relevant cost-effectiveness thresholds [36-38] using incremental cost-effective ratio decision rules and net benefit decision rules where appropriate [39].

Data on costs and resource use will be collected from Hearing Dogs for Deaf People (via structured interview and documentary analysis) and study participants at each data collection point (using a previously developed service and resource use questionnaire that will be updated specifically for this project). Costs will be calculated by applying unit costs to resource use. National unit costs (eg, the Personal Social Service Research Unit’s unit costs of health and social care) [40] will be used, where available, to aid the generalizability of findings. The cost of the service will be calculated and reported as an average cost of a hearing dog user.

For outcomes, data on health-related quality of life will be collected using the EQ-5D-5L questionnaire at multiple time points. SC- and H-QALYs will be estimated using these data. H-QALYs will be calculated using the EQ-5D-5L score [41] and the area under the curve (AUC) method [39]. The SC-QALYs will be derived by converting the EQ-5D-5L’s answers into EQ-5D-3L’s [42], as per the current recommendation by the National Institute for Health and Care Excellence [43], and then by applying the exchange rate proposed by Stevens et al [44] to obtain A Severity Characterization of Trauma (ASCOT) score to be used for the AUC method.

A regression analysis will be undertaken to account for any baseline differences in study participants between the trial arms using appropriate techniques to account for non-normality of outcomes and costs data [45]. Decision uncertainty will be addressed using probabilistic sensitivity analysis, and deterministic sensitivity analysis will be used to examine the impact on results of varying relevant parameters and assumptions.

### Nested Qualitative Study

The qualitative study seeks to understand the *active ingredients* of a hearing dog partnership, outcomes of partnerships (positive and negative, expected and unanticipated), the processes by which changes in outcomes (or not) are perceived to occur, and views on the process by which partnerships are created and supported.

It will include a longitudinal study of 15 study participants who have received their hearing dog. This number provides pragmatic balance, allowing both for (1) recruitment to cover the range of variables in the purposive sampling frame (see below) and (2) the capacity and resource demands of the longitudinal approach. A purposive sampling frame will be used (based on, eg, age, gender, age at onset of hearing impairment, presenting health and social needs, family composition, and previous experience of a dog as a pet) to ensure a range of factors and circumstances are represented. Face-to-face interviews will take place approximately 4 and 10 months post receipt of a hearing dog. Participants will be able to participate in English or BSL. Depending on the language used, we will seek permission to audio or video record the interview. For interviews conducted in BSL, a detailed summary of the interview will be produced by the interviewer, an approach we have used successfully in the past [46]. Interviews will explore experiences of the introduction of the hearing dog into the household, perceived impacts on self and wider family, views on factors that have supported or hindered the development of the partnership and its potential impact, and views and experiences of the application and matching process. During their second interview, recipients with a resident partner will be asked for permission to approach them regarding participation in a study interview. Thus, up to 15 partners (permanently living in the same household) will be recruited. Telephone or face-to-face interviews will be used to explore experiences of the introduction of the hearing dog into the household, perceived impacts (positive and negative) on self and hearing dog recipients and on the wider family, views on factors that have supported or hindered the development of the partnership and its potential impact, and experiences of the application and matching process.

A cross-sectional qualitative study will explore the views and experiences of Hearing Dogs for Deaf People staff regarding factors that hinder or facilitate positive outcomes of the hearing dog partnership. Focus groups (n=7; 5-10 participants per group) will be used to gather views and experiences of the three groups of staff most involved with hearing dog applicants and recipients: Client advisors (who support an applicant through to their *match* with a hearing dog, n=1 focus group); dog trainers (involved in matching and handover of the hearing dog to a new recipient, n=2 focus groups), and partnership instructors (responsible for ongoing recipient support, n=4 focus groups). The focus group with client advisors will explore preparation of applicants for a hearing dog, applicants’ expectations and concerns, and factors affecting efficiency and quality of the matching process. Focus groups with trainers will explore dog and person characteristics, early indicators of a *successful match* and the *active ingredients* of the intervention. Focus groups with partnership instructors will explore factors that hinder or facilitate positive outcomes of hearing dog partnerships, the maintenance of the partnership, what constitute the *active ingredients* of the intervention, and experiences of interfacing or collaborating with statutory services. The number of focus groups per staff group reflects differences in size of each workforce. Focus groups for trainers and partnership instructors will take place in both of Hearing Dogs for Deaf People’s training bases (South and North England). Overall, the number and location of groups will allow the research team to collect data of sufficient breadth and depth.

The data will be analyzed in two ways. First, for the interviews with recipients, we will create a narrative account that records their experiences of a hearing dog partnership and traces changes in outcomes and life situations of interviewees’ lives, which are in some way attributed to the partnership, and the factors that were perceived to precipitate, support, or hinder those changes. These narratives will then be collectively interrogated using thematic analytical techniques to identify and describe the *active ingredients* of a hearing dog partnership and the factors that support and hinder the effectiveness of that partnership. We have used this approach to analyzing longitudinal interviews in the past and found it a very effective and efficient tool [47].

Second, thematic analysis [48] of interviews with recipients, their partners, and Hearing Dogs for Deaf People staff will be used to identify and describe views and experiences regarding the process of creating and supporting partnerships, impacts and consequences, factors perceived to facilitate or hinder positive outcomes, and views regarding the *active ingredients* of a hearing dog partnership. An additional theme—perceived impacts on the self—will be explored for the partner interviews. For the analysis, we will use the Framework approach [49], which facilitates systematic data management and an audit trail of the analytical process.

### Patient and Public Involvement

A user advisory panel (UAP) comprising 10 individuals with a hearing dog has been formed. The panel will meet virtually using an online forum and group email. We will seek the views of the UAP on all aspects of the project, particularly the design and content of study information and consent materials, analysis of qualitative data, interpretation and synthesis of findings, and the dissemination strategy. Discussions with the UAP will be shared with the study steering committee. Hearing Dogs for Deaf People will also be consulted about dissemination and impact pathways, and we will advise them regarding their own dissemination of study findings.

### Independent Oversight of the Study

A study steering committee has been appointed comprising academics, researchers, and hearing dog recipients. The steering group will meet on four occasions over the course of the study.

### Ethics and Dissemination

The study protocol (v3 12/02/2019) included the original application and subsequent amendments (as required) received a favorable ethical opinion from the University of York Department of Social Policy and Social Work Research Ethics Committee (SPSW/S/17/1).

The study has been designed so that no participant will wait longer to receive a hearing dog than is usually expected. The nature of the two arms and the process of randomization will be made clear in study information materials as will the fact that participation is voluntary and choosing not to participate will not affect their application to Hearing Dogs for Deaf People or the service they receive. Study information materials will be developed in consultation with the user advisory panel.

### Consent

All participants will be required to provide written, informed consent on joining the study. Separate consent will be obtained for the nested qualitative study. Participants will be informed they can withdraw from the study at any time without this influencing their application.

### Confidentiality

All study-related information will be stored securely at the University of York. Participant information will be stored in locked filing cabinets in areas with limited access. All outcomes data will be anonymized and given a coded ID. Records that contain names or other personal identifiers such as address and consent forms will be stored separately from outcomes data. Electronic data will be saved on a secure university filespace, access restricted to members of the research team that have a role analysis. Following the study, all data will be archived and stored in accordance with the University of York guidelines.

### Protocol Amendments

All amendments to the protocol will be approved by the research ethics committee.

### Dissemination Policy

A summary of the findings will be published by the National Institute of Health Research’s (NIHR) School for Social Care Research. Study findings will also be reported in open access, peer-reviewed journals and at relevant conferences. Participants will be sent a summary of the findings.

### Monitoring

#### Data Monitoring

Given the nature of the trial, the trial does not have a data monitoring committee.

#### Adverse Events

The study will record and report any details of serious adverse events.

#### Auditing

This trial does not have an audit procedure in place.

#### Access to Data

The trial manager (LS) will oversee access to data. Cleaned data sets will be shared with the PI (BB), supervising statisticians (CF and CH), and economic team (HW, SW, and FL). Data dispersed to the research team will be blinded of any identifying participant information.

#### Ancillary and Posttrial Care

Not applicable for this study.

## Results

The study is funded by an NIHR School for Social Care grant, and recruitment commenced in March 2017. Recruitment is now complete, and 165 participants were randomized. Data collection is ongoing.

## Discussion

This is the first time an RCT design has been used to evaluate the impacts of the hearing dog partnership. The findings from this study will provide, for the first time, strong and reliable evidence on the impact of having a hearing dog on people’s lives in terms of their quality of life, well-being, and mental health.

In addition, we have shown that it is possible to do research that collects robust data on the impacts of assistance dogs on people’s lives. We think this study will encourage further research in this area, including for other types of assistance dogs.

The study findings will be of relevance to people with hearing impairment, Hearing Dogs for Deaf People and its supporters, and statutory services responsible for the care and support of people with hearing impairments (eg, audiology services and social work or social care services). Findings will support evidence-informed policy making, service development, and practice. Study findings will also be relevant to assistance dog organizations, in the United Kingdom and elsewhere.

## Appendix

Multimedia Appendix 1 Peer-review reports from the National Institute for Health Research - Part 1.

Multimedia Appendix 2 Peer-review reports from the National Institute for Health Research - Part 2.

Multimedia Appendix 3 Peer-review reports from the National Institute for Health Research - Part 3.

## Abbreviations

ASCOT: A Severity Characterization of Trauma
AUC: area under the curve
BSL: British Sign Language
CHE: Centre for Health Economics
EQ-5D-5L: EuroQol 5 Dimensions
GAD: Generalized Anxiety Disorder
H-QALY: health quality-adjusted life year
NIHR: National Institute of Health Research
PHQ-9: Patient Health Questionnaire-9
RCT: randomized controlled trial
SPRU: Social Policy Research Unit
SC-QALY: social care quality-adjusted life year
SWEMWBS: Short Warwick-Edinburgh Mental Well-Being Scale
UAP: user advisory panel
WSAS: Work and Social Adjustments Scale
YTU: York Trials Unit
