# Supplementary material for: Effectiveness and Cost-Effectiveness of Receiving a Hearing Dog on Mental Well-Being and Health in People With Hearing Loss: Protocol for a Randomized Controlled Trial
Source: JMIR Res Protoc. 2020 Apr 17;9(4):e15452. doi: 10.2196/15452 (PMC7195660; doi:10.2196/15452)
Supplement: Multimedia Appendix 1 [file resprot_v9i4e15452_app1.pdf]

NATIONAL INSTITUTE FOR HEALTH RESEARCH
SCHOOL FOR SOCIAL CARE RESEARCH

FULL RESEARCH PROPOSALS
Reviewer Response Form

Proposal Title: Partnerships between deaf people and hearing dogs: a mixed methods realist evaluation

PI/Lead applicant: Professor Bryony Beresford

Please note that we are likely to share comments with applicants but will only send anonymised ones.

Please provide comments on the proposal and indicate to what extent the proposal meets the criteria listed. Please refer to the guidance on completing the form.

Please note that we ask people to comment from the perspective of their experience (as an academic, service user, carer, practitioner etc.) and that various reviews/perspectives are requested for as rounded a review of research proposals as possible. If you feel unable to comment on parts of this review form, that is fine.

**Criteria**
**1. The proposal presents plans for research that has the potential to contribute to the evidence to help improve adult social care practice in England.**
The research project is relevant to the personalisation agenda within social care and the aim of enhancing well-being and social inclusion. The research has the potential to better inform those professionals and other relevant constituency groups who would have an interest in the outcomes of this particular research project which focuses on a discrete and complex form of support. The study will examine the outcomes, costs and user and service provider experiences. Although this project is not mainstream and will be carried out at a time when social services support is becoming increasingly constrained the research findings will ensure that professionals should be more confident in being able to advise people with severe or profound deafness about the service and its potential benefit for an individual (if that is what the study demonstrates). Hearing Dogs for Deaf People will also benefit from an improved evidence-base on the impact and cost-effectiveness of the service.

**2. The proposal presents research with a clear focus/question.**
The focus of the proposed study will be a 'robust and comprehensive evaluation' of hearing dog partnerships, examining the outcomes, costs and user and service provider experience. It will look at the interface between the intervention and services and support provided by statutory agencies. The focus of the proposal appears clear.

**3. The proposal has clear aims and objectives with a methodology that is appropriate to meet them.**
The need for/benefit of the research project is described. The aims and objectives are clear and set out in detail. The study design appears appropriate excepting my comments at 4. The project has been developed in partnership with Hearing Dogs for the Profoundly Deaf (HDfPD) and a hearing dog recipient but the actual methodology would be agreed primarily between the academic team. If I can make any comment, I would suggest the team have concentrated on the detailed methodology within the proposal rather than exploring more the human factors and subjects of the research.

**4. The proposal contains plans for appropriate involvement of users, carers and practitioners that will be supported and suitably resourced.**
In my view the user/carer involvement could be strengthened. I note the involvement of HDfPD and a recipient in the design of the project and also plans to set up a User Advisory Panel. There is no mention of carers being an active element in this research proposal. Although presumably the majority of the profoundly hearing impaired may well not need carer support or only minimal support, the research does not mention inclusion of carers/family. Under the heading 'Background to the study', 'The need for better evidence' it states: *'Having a hearing dog is one of the support options available. However, it requires considerable investment – both on the part of the HDfDP and* the *individual* ***(and their family)*** *– in terms of money and time'*.<br><br>I would have thought it would be essential to include input from carers/family within the design of this project, perhaps via focus groups. These carers or family members could provide valuable insight into several aspects of the hearing dog service, both around the perceived benefits to both the user and/or family members and friends around them and also the practical considerations relating to the service

**5. The proposal demonstrates good understanding of the main ethical issues likely to be involved and has appropriate ethics and research governance measures.**
The research ethics and governance issues and processes appear appropriate.

**6. Understanding of, and commitment to equality issues as relevant to the research – SSCR wishes its research to be as inclusive as possible, including (where possible) of people who lack capacity to consent. Has the proposal set out to be as inclusive as possible, including suitable resourcing?**
This is a specialist project focusing on the profoundly hearing impaired. The selection of individuals on the advisory group should bear diversity in mind when recruiting members. The description of recruitment to the randomised controlled trial of individuals to the study does not mention issues of inclusion relating to social group, ethnicity etc. Perhaps this may be considered and could be monitored by the advisory group.

7. Plans for the communication of research and building to maximise impact from the work, both through traditional publication routes and directly to audiences of policymakers, practitioners and service users and other forms of engagement.

The plans for maximising impact and knowledge exchange appear appropriate to the task. As an emotive subject area is would be beneficial to encourage some press interest in relating to the wider public interest and raising the profile of deaf people in society.

8. Does the proposal represent good value for money?

I have no comment on the budget headings excepting the resource requested for user/carer involvement of £300. This figure appears low to me and in comparison with the other resource indicators appears somewhat out of proportion. Perhaps the same can be said for practitioner involvement.

9. Expertise of the team

This appears appropriate

10. Any additional comments and overall assessment of the proposal you may have.

The hearing dog service require considerable investment in money and time. It is appropriate that a robust evaluation of the service is carried out.

Thank you for your help in doing this review. It is much appreciated by everyone at the NIHR School for Social Care Research.
