# Supplementary material for: Effectiveness and Cost-Effectiveness of Receiving a Hearing Dog on Mental Well-Being and Health in People With Hearing Loss: Protocol for a Randomized Controlled Trial
Source: JMIR Res Protoc. 2020 Apr 17;9(4):e15452. doi: 10.2196/15452 (PMC7195660; doi:10.2196/15452)
Supplement: Multimedia Appendix 2 [file resprot_v9i4e15452_app2.pdf]

NATIONAL INSTITUTE FOR HEALTH RESEARCH
SCHOOL FOR SOCIAL CARE RESEARCH

FULL RESEARCH PROPOSALS
Reviewer Response Form

Proposal Title: Partnerships between deaf people and hearing dogs: a mixed methods realist evaluation

PI/Lead applicant: Professor Bryony Beresford

Please note that we are likely to share comments with applicants but will only send anonymised ones.

Please provide comments on the proposal and indicate to what extent the proposal meets the criteria listed. Please refer to the guidance on completing the form.

Please note that we ask people to comment from the perspective of their experience (as an academic, service user, carer, practitioner etc.) and that various reviews/perspectives are requested for as rounded a review of research proposals as possible. If you feel unable to comment on parts of this review form, that is fine.

**Criteria**
**1. The proposal presents plans for research that has the potential to contribute to the evidence to help improve adult social care practice in England.**
The research is relevant to current practice in adult social care in England and is likely to result in improvements in practice possibly over the next 3 to 4 years.<br>This application also has the potential to further use of trials in under evaluated areas of social research.<br><br>Use of HDfDP comes with a fairly considerable cost in terms of training and support and a detailed analysis of whether and how this programme works is useful to social care research and practice.

**2. The proposal presents research with a clear focus/question.**
Yes, the research question is clear.

**3. The proposal has clear aims and objectives with a methodology that is appropriate to meet them.**

The overarching aims of the research proposal are clear but more clarity would be beneficial in terms of what questions will be answered.

The need for the research is clearly outlined but the proposal becomes a bit unclear when describing the RCT. It is not clear when each of the outcome measures are being used (i.e. at which time points). It is also not clear what is meant by an 'early recruit' and whether there is a cut off for this.

What are the recruitment processes? Where will individuals be recruited from? Does geography have an impact in the recruitment process?

What information will be provided to the 'hidden group'. From the description it sounds as though they will just be on a waiting list for matching during this time. Would it be appropriate for them to have some sort of support or contact with the team during this time? Have the team considered what will happen if individuals can't be matched within the specified time frames?

Mediating and moderating factors are mentioned in the aims – how are the team going to test for these?

The qualitative study is a good inclusion. It is not clear what information will be gathered from the Sensory Impairment Teams in the interviews.

Have the study team considered over recruiting and if this will be an issue? The sample size calculation does not seem to be based on any previous research or evidence.

Does the team plan to examine the results by age or gender?

4. The proposal contains plans for appropriate involvement of users, carers and practitioners that will be supported and suitably resourced.

Yes it is clear that people with appropriate individuals as well as other stakeholders have been and will continue to be heavily involved in this project in an appropriate way.

5. The proposal demonstrates good understanding of the main ethical issues likely to be involved and has appropriate ethics and research governance measures.

Yes, a clear understanding of governance and ethical issues has been included.

6. Understanding of, and commitment to equality issues as relevant to the research – SSCR wishes its research to be as inclusive as possible, including (where possible) of people who lack capacity to consent. Has the proposal set out to be as inclusive as possible, including suitable resourcing?

Yes, the proposal does not indicate any groups will be excluded from the research.

7. Plans for the communication of research and building to maximise impact from the work, both through traditional publication routes and directly to audiences of policymakers, practitioners and service users and other forms of

engagement.
Will the study team report the findings back to participants at the end of the trial and how will this be done? There is an adequate mixture of both academic dissemination activities and those aimed at professionals, service providers and policy makers.

8. Does the proposal represent good value for money?
All cost requests appear to be appropriate and necessary.

9. Expertise of the team
I have no concerns about the expertise of the team.

10. Any additional comments and overall assessment of the proposal you may have.

Thank you for your help in doing this review. It is much appreciated by everyone at the NIHR School for Social Care Research.
