# Supplementary material for: Effectiveness and Cost-Effectiveness of Receiving a Hearing Dog on Mental Well-Being and Health in People With Hearing Loss: Protocol for a Randomized Controlled Trial
Source: JMIR Res Protoc. 2020 Apr 17;9(4):e15452. doi: 10.2196/15452 (PMC7195660; doi:10.2196/15452)
Supplement: Multimedia Appendix 3 [file resprot_v9i4e15452_app3.pdf]

NATIONAL INSTITUTE FOR HEALTH RESEARCH
SCHOOL FOR SOCIAL CARE RESEARCH

FULL RESEARCH PROPOSALS
Reviewer Response Form

Proposal Title:

PI/Lead applicant:

Please note that we are likely to share comments with applicants but will only send anonymised ones.

Please provide comments on the proposal and indicate to what extent the proposal meets the criteria listed. Please refer to the guidance on completing the form.

Please note that we ask people to comment from the perspective of their experience (as an academic, service user, carer, practitioner etc.) and that various reviews/perspectives are requested for as rounded a review of research proposals as possible. If you feel unable to comment on parts of this review form, that is fine.

**Criteria**
**1. The proposal presents plans for research that has the potential to contribute to the evidence to help improve adult social care practice in England.**
The research appears to be quite relevant to inform current practice in adult social care in England and provide fresh evidence about under researched issues related to hearing dog partnerships, whist covering two separate methodological and applied streams. However it is not clear how the results from the two separate streams (in particular the methodological one) will inform/improve current practice. Practical examples of how the results could be used to support possible methodological developments and policy shifts (and over what timeframe) would be very useful.

**2. The proposal presents research with a clear focus/question.**
I think there are two main separate questions (methodological and practical) that would need to be both presented and addressed throughout the proposal. The main focus of the proposal is on the evaluation of the intervention whereas more information would be needed to: (i) explain also the methodological development of the complex intervention prior to its actual evaluation; (ii) the impact of both the methodological and practical elements of the research.

**3. The proposal has clear aims and objectives with a methodology that is appropriate to meet them.**

Aims and objectives need to be both clearly stated (aims are currently missing). The list of objectives provided so far cover 8 separate items that would need to be revised and prioritised according to primary and secondary objectives.

Methodological development of the intervention - I would appreciate to know more about the development of the intervention and possible framework that would be used to inform the process (e.g. MRC framework for complex intervention?) and reference to possible piloting and feasibility work to be confident that the intervention presented here can be delivered as intended.
More info is also needed about how the research findings would aim to contribute to the methodological debates re complex interventions and the practical implications of this project.

Quantitative valuation of the intervention –further clarification is needed re the design of the trial (eg the choice of the comparative group), why the trial and the economic evaluation would need to be separate exercises and how the trial data would inform the economic evaluation. The economic evaluation would compare the hearing dog practice with usual care (still to be defined?). It is not clear from where data would be sourced (the trial participants and HDfDP? what about usual care data?), the type of economic data collected across the different sources, whether they would adopt a prospective/retrospective data collection approach, the sample size and the timeframe used.

4. The proposal contains plans for appropriate involvement of users, carers and practitioners that will be supported and suitably resourced.

I would suggest to expand the user involvement to include also the piloting and testing of the quantitative tools as well as discussion around the analysis of the quantitative data and dissemination of the results.
The stakeholder involvement in the study is limited to a group of individuals with hearing dogs (see UAP); added benefit could be derived from the creation of a steering group committee including the participation of both users and representatives from HDfDP, social care practitioners, and policy makers that would meet at key stages during the course of a project and influences strategic decisions.

5. The proposal demonstrates good understanding of the main ethical issues likely to be involved and has appropriate ethics and research governance measures.

I am worried that the current ethic application plan may be too ambitious as the team would aim to complete all the preparatory work and gain ethical approval within the first three months. All the paperwork (eg protocol, data collection tools, information letters, consent forms etc) to be submitted as appendices to the actual ethical application forms should be developed and finalised with the relevant stakeholder groups. This would reassure the relevant committees that the different stakeholder groups have been involved in the process and the project addresses possible issues and concerns raised by them.

**6. Understanding of, and commitment to equality issues as relevant to the research – SSCR wishes its research to be as inclusive as possible, including (where possible) of people who lack capacity to consent. Has the proposal set out to be as inclusive as possible, including suitable resourcing?**
I did not capture any obvious gaps in their plans for inclusion that would undermine any findings from the project.

**7. Plans for the communication of research and building to maximise impact from the work, both through traditional publication routes and directly to audiences of policymakers, practitioners and service users and other forms of engagement.**
The list of outputs and proposed knowledge exchange activities are limited to written communications to be disseminated at the end of the study. It would be very useful to integrate the current communication plan with additional events (e.g. face-to-face gatherings supported by BSL interpreters to be run at key stages of the research process, presentations to relevant stakeholders associations and groups) to allow additional interaction of the different stakeholder groups with the team, maximise their contribution to the research and knowledge exchange between the parties.

**8. Does the proposal represent good value for money?**
The research team appears to have the right skills and experience to carry out the research. However the requested level of resource should be amended to address a lack of user/carer/practitioner involvement throughout the development of the research process and final impact activities (eg see steering group meetings and presentations to relevant stakeholders associations and groups).<br><br>I am worried that the project plan timetable is too ambitious, in particular regarding the development of the project material (including the different data collection tools for qualitative and quantitative analyses) and the ethical approval process.

**9. Expertise of the team**
The applicants appears to have the level of expertise and experience to give confidence that they can successfully complete the proposed project with the support of relevant stakeholder group representatives as needed (eg see the creation of steering group as suggested above).

**10. Any additional comments and overall assessment of the proposal you may have.**

Thank you for your help in doing this review. It is much appreciated by everyone at the NIHR School for Social Care Research.
